# Serum VEGF Level Is Different in Adolescents Smoking Waterpipe versus Cigarettes: The Irbid TRY

**DOI:** 10.3390/biom8040102

**Published:** 2018-09-28

**Authors:** Mahmoud A. Alomari, Nihaya A. Al-Sheyab, Omar F. Khabour, Karem H. Alzoubi

**Affiliations:** 1 Division of Physical Therapy, Department of Rehabilitation Sciences, Jordan University of Science and Technology, Irbid 22110, Jordan; 2 Division of Physical Education, Department of Educational Sciences, Qatar University, Doha 2713, Qatar; 3 Faculty of Applied Medical Sciences, Allied Medical Sciences Department, Jordan University of Science and Technology, Irbid 22110, Jordan; nasheyab@just.edu.jo; 4 Faculty of Nursing, Maternal and Child Health Department, Jordan University of Science and Technology, Irbid 22110, Jordan; 5 Department of Medical Laboratory Sciences, Jordan University of Science and Technology, Irbid 22110, Jordan; khabour@just.edu.jo; 6 Department of Clinical Pharmacy, Jordan University of Science and Technology, Irbid 22110, Jordan; khalzoubi@just.edu.jo

**Keywords:** vascular endothelium growth factor, waterpipe cigarette, tobacco, Irbid-TRY

## Abstract

Waterpipe (Wp) use is associated with most devastating diseases and particularly popular among adolescents. Vascular endothelium growth factor (VEGF) is essential for generating new vessels. The effect of smoking tobacco on VEGF is controversial and unknown among adolescents. Therefore, the current study compared serum VEGF in adolescents smoking cigarettes (Cg) only (9.3%), Wp only (19.6%), and dual (Wp and Cg) (36.4%) versus nonsmokers (34.6%) in adolescents. A self-reported questionnaire and enzyme-linked immunosorbent assay (ELISA) were used to obtain smoking status and serum VEGF, respectively, in 475 (age: 14.6 ± 1.0 years) boys (*n* = 263) and girls (*n* = 212) from Irbid, Jordan. The analysis showed that smoking status (R^2^ = 0.021; *p* = 0.001) and gender (R^2^ = 0.035; *p* = 0.000) can predict VEGF. Furthermore, 2-way-ANCOVA revealed that VEGF was lower in the dual cohort versus the Cg (33.4%; *p* = 0.04) and nonsmoker (29.6%; *p* = 0.003) cohorts; VEGF in smokers, was lower (33.6%; *p* = 0.04) in the Wp versus nonsmokers in the boys but not the girls. These results are unique and suggest that smoking lowers VEGF, which might adversely affect vascular growth and function. This is alarming given that adolescents are still in the development stage and smoking, particularly Wp, is popular among them. Therefore, interventions targeting smoking among schoolchildren are urgently needed to avoid the negative effects of smoking, especially on vascular health.

## 1. Introduction

Smoking tobacco is associated with the leading causes of hospitalization, morbidity, and mortality [1,2]. Among adolescents, it is associated with altered cardiovascular [3,4], respiratory [5], hormonal [6,7], and musculoskeletal [8] functions. Waterpipe (Wp) is a tobacco consumption style that involves inhaling smoke passing through a bowl of water then a hose into the smoker’s mouth. It has recently reemerged as a favorable tobacco consumption style [9], especially among adolescents [10,11,12]. It is currently popular in Europe [13], USA [14], Canada [15], and Australia [15,16]. In fact, Wp smoking has exceeded that of cigarettes [9,11,12]. Factors that make Wp attractive include its aroma, flavor, and social gathering aspect [9]. Smoking Wp is associated with numerous adverse health effects [17], and has been linked to the most devastating diseases, including cardiovascular [18] and respiratory diseases [19], stroke [20], and cancer [21].

Vascular endothelial growth factor (VEGF) is a multifunctional/organ cytokine glycoprotein produced mainly in the vascular endothelium [22]. Additionally, the lungs, kidneys, heart, adrenal glands, retinal pigment epithelium, pericytes, T cells, and macrophages are also potential production sites [22]. Deficiency in O_2_, glucose, and inflammatory reactions are the primary stimulants for VEGF synthesis [22]. The physiological function and clinical importance of VEGF are diverse. VEGF is a potent mitogen particularly important for angiogenesis and lymphogenesis in health and disease [22]. It functions mainly in vascular endothelial cells to promote healthy pre- and postnatal vessel proliferation. VEGF is involved in the vascularization of the embryo, female reproductive tract, and is active during wound repair [22]. Most importantly, it is pivotally involved in coronary and peripheral arterial proliferation, indicating a therapeutic effect for revascularization during vascular ischemia and stenosis [23]. 

VEGF is also involved in most devastating disorders, cardiovascular diseases, and cancer. It plays a pivotal role in atherogenesis [24] and tumorigenesis [25]. It locally initiates blood vessel permeabilization for the penetration of plasma proteins and stromal cells [24]. Additionally, it promotes the sprouting of new blood vessels that supply tumor cells with O_2_ and nutriments to facilitate metastasis [25]. These involvements suggest a therapeutic potential could be exerted by inhibiting VEGF action at these pathological sites [25]. 

The effect of smoking among adults is controversial. For example, in a study conducted on young adults, 30 min of acute secondhand smoke exposure resulted in an immediate increase in VEGF that lasted for 24 h [26]. An increase in VEGF was also observed in healthy male, but not female [27] smokers. Additionally, smokers with ankylosing spondylitis were more likely than nonsmokers [28] to be affected by genetic polymorphisms [29]. However, similar plasma/serum VEGF levels were reported in healthy adult smokers and nonsmokers [30,31,32]. Conversely, exposure to cigarette (Cg) smoke under hypoxia resulted in inhibiting in vitro/vivo angiogenesis subsequent to reduced VEGF expression [28]. In addition, VEGF expression was lower in the neutrophils of smokers than in nonsmokers [33]. These studies demonstrate gaps in the literature and that the relationship between smoking and VEGF among adults is complex and might be affected by gender and/or health status. The aim of the current study is to compare serum VEGF levels in adolescents smoking Cg only, Wp only, and dual (concurrent Wp and Cg) versus nonsmokers. Since the adolescent stage is characterized by rapid growth, we hypothesized that the relationship between VEGF and smoking might be different than that reported during adulthood. In addition, the toxicant profiles of Cg and Wp have been shown to be different. For example, Wp smoke contains high levels of polyaromatic hydrocarbons (PAHs), volatile aldehyde, and CO than that of Cg smoke [34], which might modulate VEGF expression [35,36,37,38,39]. Furthermore, compared to Cg, additional toxicants are derived from charcoal, flavors, added sugars, and glycerol that are in Wp smoke [40]. Thus, the effects of Wp and Cg on VEGF expression levels might be different. The results will further enhance the knowledge of the relationship of smoking with VEGF. Additionally, they will further help understanding the adverse health effects of Wp, particularly among adolescents, and subsequently determine the therapeutic potential of VEGF.

## 2. Materials and Methods 

### 2.1. Design and Recruitment

The current manuscript used a descriptive and cross-sectional design to compare serum VEGF in adolescents smoking different types of tobacco (Cg only, Wp only, and both Cg and Wp) versus adolescents who have never tired any type of tobacco (nonsmokers). The data were derived from the “Irbid Tobacco Risk in Youth (Irbid-TRY)” longitudinal project. The sampling plan and participants’ recruitment were detailed elsewhere [3,11,41]. A total of 475 adolescents with an age range of 12 to 17 years were recruited to partake in the study.

All adolescents voluntarily provided written consent to participate after obtaining written parental informed consent. The Institutional Review Board of Jordan University of Science and Technology and the Jordanian Ministry of Education approved the study protocol and processes. The Institutional Review Board number is MA/214/2017. Sociodemographic characteristics, tobacco smoking patterns, and blood samples were taken from all participating adolescents, as described below.

The adolescent sociodemographic characteristics were obtained using self-reported questionnaires. Additionally, standared tape-measure and digital weighing scales were used to determine height and weight, respectively. Subsequebtly, Body Mass Index (BMI) was calculated using the standard equation: BMI = height/(weight)^2^.

### 2.2. Smoking Status

A self-reported Arabic language-validated survey was used to obtain tobacco smoking status [42,43]. Smoking patterns include the four categories of smoking (ever use, current use, regular use, and dual use). ‘Ever use’ is the use of smoking anytime in the past; at least once in a lifetime. In the survey, two separate questions asked about the ever use of either Wp or Cg smoking. Additionally, two similar questions were asked in the survey about Wp and Cg smoking patterns. Students were only considered current Waterpipe only smokers when they reported Wp smoking but not any other type of tobacco consumption in the past month [42]. The same definition applies to current Cg smoking, while students who reported both Cg and Wp smoking in the past month were considered dual smokers. Regular tobacco (Wp, Cg, or dual) smoking was defined as smoking at least once within the last week. 

### 2.3. Blood Sampling

Fasting blood samples were drawn from participants’ antecubital veins into plain glass tubes while they were in a sitting position. After 15 min of collection, samples were centrifuged for 8–10 min at 1500× *g* to obtain serum. Serum samples were then divided into several aliquots and immediately stored at −80 °C [44,45].

### 2.4. Vascular Endothelium Growth Factor

Serum VEGF levels were determined using the enzyme-linked immunosorbent assay (ELISA), specific for VEGF according to the kit manufacturer’s instructions (Human VEGF, DuoSet ELISA Kit; R&D Systems, Minneapolis, MN, USA). Plates were read at the kit’s specified wavelength using an automated reader (Epoch Microplate Spectrophotometer, Bio-tek instruments, Highland Park, Winooski, VT, USA). VEGF levels were calculated based on the values of the standard supplied with the kit. Samples from each group were included in every ELISA plate [46]. 

### 2.5. Statistical Analysis

Statistical analyses were accomplished with SPSS software for Windows (version 22.0; Chicago, IL, USA). Data are expressed as means ± SD, and α was preset at *p* < 0.05. A group of simple linear regressions were used to determine the individual relationship of VEGF with smoking status (i.e., nonsmokers versus Cg only, Wp only, and dual smoking), gender, age, location (rural versus urban), and BMI. Subsequently, a stepwise regression was used for the factors was significantly related to VEGF in the simple linear regression to predict the VEGF level in participating adolescents. One and two way ANCOVA were used to examine the differences in VEGF levels. Additional LSD posthoc comparisons were used to determine differences between specific groups.

## 3. Results

A total of 2691 adolescents participated in the study, of which serum VEGF was obtained from 608, while smoking status was obtained from 2445 adolescents. The total adolescents with smoking and VEGF information was 475. As shown in Table 1, among the adolescents, 212 were girls, 314 were normal weight, 139 were in the 8th grade, and 173 were dual smokers. 

As in Table 2, the simple linear regression showed that smoking status (F = 10.20; *p* = 0.001) and gender (F = 4.783; *p* = 0.029) predicted 2.1% and 0.8% of the VEGF level. However, VEGF was not related to age (F = 1.564; *p* = 0.212), height (F = 2.82; *p* = 0.094), weight (F = 0.600; *p* = 0.44), percent body fat (F = 0.987; *p* = 0.321), waist circumference (F=1.075; *p* = 0.300), or BMI (F = 1.16; *p* = 0.282). Stepwise regression, as shown in Table 2, which included smoking status (R^2^ = 0.021; *p* = 0.001) and gender (R^2^ = 0.035; *p* = 0.000), revealed that both were related to VEGF. 

**Figure 1:**
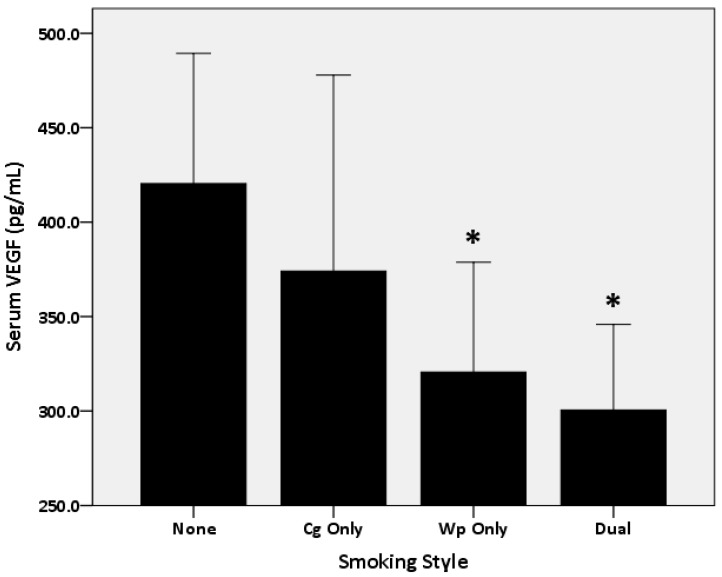
Comparison in serum vascular endothelium growth factor (VEGF) level among the Adolescents Smoking Cigarette Only, Waterpipe Only’ and Dual Smokers groups versus nonsmokers (None). * *p* > 0.05 versus nonsmokers.

**Figure 2:**
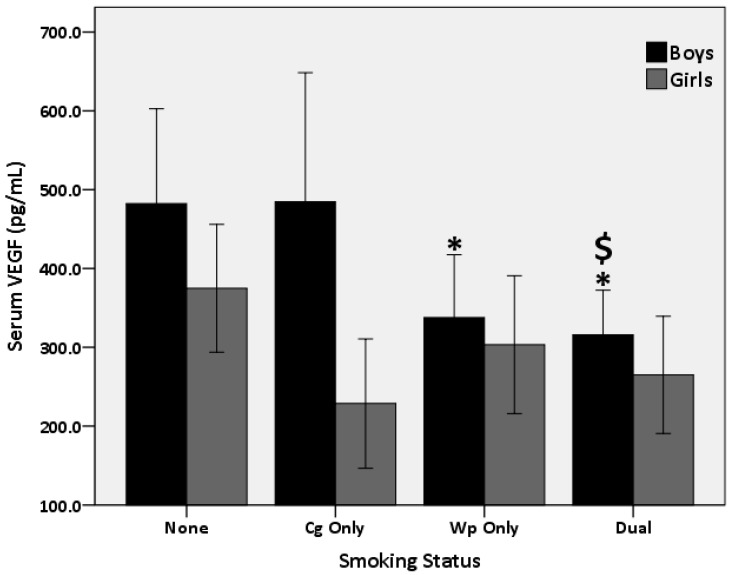
Comparison in serum VEGF levels between boys and girls Smoking Cigarette Only, Waterpipe Only, and Dual Smokers versus nonsmokers (None). * *p* > 0.05 versus nonsmokers. ^$^ *p* < 0.05 versus Cigarette Only.

As in Figure 1, the 1-way ANCOVA analysis, after covariating for age and BMI, showed a main effect for smoking status (*p* = 0.02). Posthoc comparisons showed lower VEGF in the Wp (*p* = 0.04) and dual (*p* = 0.004) versus the nonsmokers, no difference (*p* > 0.05) was found between the Wp and dual smokers or between the nonsmokers and Cg smokers. As in Figure 2, a 2 (Gender)* 4 (smoking status) ANCOVA, after covariating for age and BMI, showed a main effect for gender (*p* = 0.005) (i.e., greater in the boys) and smoking status (*p* = 0.01), but no (*p* > 0.05) interaction effect. Additional posthoc comparisons demonstrated that VEGF was lower in the dual versus the Cg (*p* = 0.04) and nonsmokers (*p* = 0.003), while it was lower (*p* = 0.04) in the Wp versus nonsmokers without differences (*p* > 0.05) between the Wp and dual smokers or between the nonsmokers and Cg smokers in the boys only. However, the posthoc comparison showed no differences (*p* > 0.05) in VEGF between the smoking groups in the girls. 

## 4. Discussion

The study examined the relationship of smoking Cg only, Wp only, and dual (concurrent Cg and Wp) with serum VEGF among adolescents. The analysis showed that smoking status and gender can predicted VEGF. Additional comparisons demonstrated that VEGF was lower in the Wp only and dual versus the nonsmokers. Further analyses demonstrated that these differences were found only in the boys, but not in the girls. These results are unprecedented and suggest that smoking, especially Wp, is negatively associated with VEGF levels in boys. However, future cross-sectional, longitudinal, and interventional studies are needed to verify these findings. 

A few studies with conflicting results examined the effect of tobacco consumption on VEGF [26,27,28,29,30,31]. These studies reported either an increase [26,27], decrease [47], or no change [30,31,32] in VEGF concentration. Hence, these studies used Cg, but not Wp, smoking in adults, while the current study included adolescents smoking Wp, which makes the current findings unique.

Circulatory VEGF in the current study was similar among the adolescents smoking Cg versus nonsmokers confirming previous findings in adults [30,31,32]. Though these studies reported altered vascular function, measured with flow-mediated vasodilatation and thrombomodulin level, no differences were observed in VEGF among the healthy smokers versus the nonsmokers [30,31]. However, in one of the studies, an inverse relationship of VEGF with vascular function was found among the smokers suggesting a predisposition to endothelial dysfunction [30].

The differences in VEGF were found only among the Wp (i.e., Wp only and dual) smokers. This is quite interesting, yet difficult to compare as no previous studies have reported VEGF levels among Wp smokers in adolescents or otherwise. However, it seems that Wp smoke contains materials that diminishes VEGF bioavailability by either inhibiting production or increasing depletion. A previous study reported that in vitro/vivo ingestion of a Cg smoke extract resulted in diminished VEGF expression and level coupled with negatively altering angiogenesis and blood flow [47]. However, the mechanisms by which Wp smoking induces a decrease in VEGF levels need to be investigated. For example, carbon monoxide [48] and polycyclic aromatic hydrocarbons (PAH) [49] are several fold richer in Wp versus Cg smokes. Exposure to carbon monoxide has been reported to interfere with VEGF mRNA and protein induction in arterial smooth muscle [50] and umbilical vein endothelial [51] cells. Alternatively, exposing rats to diesel exhaust (i.e., rich in PAH) can induce cardiac dysfunction and decrease VEGF levels subsequent to the activation of aromatic hydrocarbon receptors [52]. A significant decrease in VEGF levels was also reported in pregnant mice [53] and in the ischemic hindlimb of mice exposed to PAH Benzo(a)pyrene [54]. Conversely, after being exposed to Benzo(a)pyrene, induction of VEGF by PAH increased in epidermal [55] and lung cancer cells [56]. These studies demonstrate differential effects of PAH on the expression of VEGF in healthy versus unhealthy cells. Finally, compared to Cg smoke, Wp smoke contains additional toxicants derived from the burning of charcoal, added flavors, glycerol, and sugar [34,57]. Therfore, the possible contribution of these toxicants to the observed differences in VEGF among Wp smokers cannot be excluded. Thus, multiple mechanisms could account for the observed changes in VEGF levels in the circulation of Wp adolescent users, which warrants more studies to understand these mechanisms.

The study suggests gender differences in VEGF among Wp smokers, in boys but not in girls. This is another intriguing but unexplainable finding, as no studies have reported these results in adolescent Wp smokers. However, maybe the boys inhale more Wp smoke or use Wp more intensely and frequently. Reports have shown that boys tend to inhale Cg smoke for greater duration and volume while exhibiting a shorter interpuff interval [58]. This means that the boys would consume more smoke than the girls [59]. Similarly, studies have reported that Cg smoking in the boys is more intense and frequent than girls, which, once again, would mean greater smoke consumption [60]. In a recent study from United States, toxicant exposure to Wp smoke was greater in men than women with men inhaling a greater smoke volume and higher plasma nicotine concentrations after smoking [61]. This greater smoke consumption indicates that the boys are more exposed to smoke toxicants, thus may be more affected. It is worth mentioning that gender differences in VEGF levels were reported in adults Cg smokers with and without diseases. For example, in a study conducted on 443 adults, the mean serum VEGF concentration of male smokers was significantly higher than that of female smokers [27]. In addition, VEGF levels were greater in male smokers with chronic obstructive pulmonary disease [62] and with Fabry disease compared to females [63]. Thus, observed gender differences in VEGF levels in Wp smokers could be related to the interactions of toxicants with some gender specific intrinsic factors. However, these are mere speculations, thus Wp smoking topography, intensity, and frequency in boys vs. the girls need to be investigated to further understand VEGF-gender differences found herein. 

Smoking Wp is rapidly spreading throughout the globe, especially among adolescents [10,11,12]. Recent studies have shown that the combined prevalence of Wp only and dual has exceeded 55% among adolescents, especially in boys [11,12]. Additionally, it has recently gained popularity in many countries outside of the Middle East, the traditional location of Wp, and has spread to Europe [13], USA [14], Canada [15], and Australia [15,16]. Additionally, studies have shown that Wp smoking has recently surpassed that of Cg [9,11,12]. This increase in prevalence is alarming and requires extra efforts to restrain the spread and offset the adverse health effects of smoking Wp among adolescents, especially in boys. 

The physiological and clinical significance of VEGF is not totally understood. However, it is well-established that VEGF plays a pivotal role in angiogenesis at various locations of the body in healthy and diseased tissues. Since the adolescents in the current study are apparently healthy without any overt signs of disease, it is most likely that VEGF is important for the function of healthy tissue. It contributes to initiating, maintenance, and repair of cardiovascular, cerebral, and peripheral vascular circuits. Therefore, the reduced VEGF found herein is most likely a negative effect of smoking Wp as it might adversely affect vascular health, function, and growth. However, more studies are certainly needed to confirm these findings and verify these speculations. 

Given the cross-sectional design of the current study, it is impossible to conclude a cause–effect relationship. It is also important to mention that the relatively smaller sample size limits the generalizability of the findings. Similarly, the study was conducted in Irbid, Jordan, which confines the implications of the results in other communities, countries, and ethnicities. In addition, the study did not collect specific details such as how many uses per day for tobacco products and time between blood sampling and last tobacco use that can be used in the interpretation of the observed variations between Cg and Wp on VEGF levels. Therefore, studies using an interventional and longitudinal design with a larger sample size from various ethnicities with the inclusion of additional parameters that measure tobacco use are needed to understand the health effects of Wp, particularly vascular health, among adolescents.

## 5. Conclusions

The study compared serum VEGF in adolescent smoking Cg only, Wp only, and dual. The results showed diminished levels of VEGF in adolescents smoking Wp only and dual, but not Cg only, versus nonsmokers, especially among the boys. Given the importance of VEGF for angiogenesis, the current results indicate that Wp smoking adversely affects vascular function. These results confirm the adverse health effects of smoking, particularly Wp. Additionally, studies are warranted to confirm these findings and verify these speculations. Furthermore, studies and programs specially designed to restrain the disproportional increase in smoking, particularly Wp among adolescents, are needed. 

## Figures and Tables

**Table 1:** The participants’ demographic characteristics (*n* = 475).

Characteristic	Value
Gender (% boys)	58.2
Age (years, mean (SD))	14.6(1.0)
Weight (kg, mean (SD))	56.4(14.1)
Height (cm, mean (SD))	160.6(8.4)
BMI (kg/m^2^, mean (SD))	21.7(4.4)
Underweight (%)	3.2
Normal weight (%)	53.5
Overweight (%)	14.8
Obese (%)	8
**Grade (%)**	
7	16.5
8	28.4
9	30
10	25.1
**Location (%)**	
Rural	57.7
Urban	42.3
**Smoking Status (%)**	
Never smoked	34.7
Cigarette smokers	9.3
Waterpipe smokers	19.6
Dual smokers	36.4

**Table 2:** Linear regression for vascular epithelium growth factor (VEGF) predictors.

	b2-Value	F-Value	95% CI	*p*-Value
Simple linear regression				
Smoking status	−40.8	10.2	−65.8/−15.7	0.001
Gender	−62.5	4.78	−118.6/−6.4	0.029
Age	−17.64	1.56	−45.4/10.1	0.212
Height	−3.27	2.819	−7.11/0.56	0.094
Weight	−0.428	0.600	−1.51/0.66	0.439
Percent body fat	−2.1	0.987	−6.203/2.03	0.321
Waist circumference	−1.56	1.075	−4.52/1.39	0.300
BMI	−4.032	1.16	−11.4/3.33	0.282
Stepwise linear regression				
Smoking status	−40.8	10.2	−65.8/−15.7	0.001
Gender	−88.5	8.59	−154.8/−22.1	0.000

BMI: Body Mass Index.
